# The characteristics of husbands and violence against women in Wuhan, China: a cross-sectional study

**DOI:** 10.1186/s12905-022-01650-z

**Published:** 2022-03-14

**Authors:** Xuening Chang, Yifan Yang, Ruizhen Li

**Affiliations:** Child Health Section, Wuhan Children’s Hospital (Wuhan Maternal and Child Healthcare Hospital), Tongji Medical College, Huazhong University of Science and Technology, Wuhan, 430000 China

## Abstract

**Objective:**

To explore the prevalence and correlation between husbands and lifetime domestic violence (DV) among women in Wuhan, China.

**Methods:**

A cross-sectional study was conducted in a community health center in Wuhan from June 2015 to December 2015. A total of 1015 women who came to the center for gynecological examination were selected through a random sampling. They were assessed using the WHO Violence Against Women Instrument to evaluate the prevalence of DV. The chi-square test, the Wilcoxon rank test, and unadjusted and adjusted logistic regression analyses were used to analyze the possible risk or protective factors for DV.

**Results:**

The lifetime prevalence of DV was 29.36% (298/1015). The risk factors included heavy physical labor (OR 3.54, 95% CI 1.63–7.77), long-term drinking (OR 1.60, 95% CI 1.19–2.14), overweight or obesity (OR 1.36, 95% CI 1.01–1.88) and long-term smoking (OR 1.03, 95% CI 1.01–1.04). Higher education was a protective factor (OR 0.80, 95% CI 0.66–0.96).

**Conclusion:**

Women whose husbands had lower education, performed heavy physical labor, were long-term alcohol consumers, had overweight or obesity, and were long-term smokers were vulnerable to lifetime DV.

## Introduction

Violent behaviors perpetrated by men against women have been an unfortunate element of the life experience of individuals in different civilizations across history. DV is often perpetrated by a husband, an ex-husband, a boyfriend, or an ex-boyfriend, so it is also referred to as intimate partner violence (IPV) [1]. IPV includes physical violence, psychological violence, and sexual violence. Ninety percent of the perpetrators were husbands [2]. The incidence of DV among women of childbearing age is concentrated at 7–35.0% in the United States and other developed countries [3–7]. In relatively underdeveloped countries such as Ethiopia (78.0%), Nigeria (35.9%), and India (41.0%), the incidence of DV is higher [8–10]. In China, the literature shows that the incidence of DV in urban areas is concentrated at 11.3–45.1% [11–13]. Under the premise of a large population base, the number of women suffering from DV is large. Influenced by traditional Chinese thinking, some women are reluctant to disclose the violence [14], which would lead to a higher incidence of actual DV.

DV is influenced by several complicated factors, including sociodemographic characteristics, behavioral habits, marital status, and so on. These factors directly or indirectly contribute to the formation of male violence in the family. Younger age, no personal income, lower female literacy, and shorter marital duration are risk factors for DV [15–17]. A husband’s low level of education, a husband who smokes, and lower family income are risk factors for DV, and a shorter marital duration is a protective factor [18]. There are regional differences in the occurrence of DV and its risk factors, which may be related to the sociocultural environment and economic status of each region. To effectively reduce the incidence of DV, it is necessary to combine local realities and reduce the various risk factors identified in local investigations.

At present, community studies of the correlation between DV and husbands’ characteristics based on female samples are rare in China [19]. Therefore, this study focused on the correlation between lifetime DV among women and husbands' demographic and behavioral characteristics to facilitate DV prevention and interventions for women in Wuhan, China.

## Methods

### Study design

A cross-sectional study was conducted in a community health center in Wuhan from June 2015 to December 2015. Participants were married, divorced, and widowed women aged 20–60 years who came to the center for gynecological examination and were selected through a random sampling method. Computer-generated random numbers were used to randomly select patients as they signed in with a nurse at the community health center if they met the study criteria and were interested in participating. Unmarried women were excluded. A total of 155 women refused to take part in the study and 1015 women participated in the investigation. The survey was conducted by trained investigators with an anonymous, self-administered questionnaire. Respondents completed the survey with the trained investigators on the research team guiding them through the process of survey completion and check-out. This study was approved by the Institutional Ethical Review Board (IRB) of the School of Public Health, Tongji Medical College, Huazhong University of Science and Technology. Informed consent was obtained from all participants. All methods in the present study were performed following the relevant guidelines and regulations of the Declaration of Helsinki.

### Variables and measuring tool

A “husband” is defined as legally married men. Partners other than legally married husbands were not included in this study. The demographic characteristics involved in the study included age, spousal educational difference, occupation, education, body mass index (BMI), monthly income, marital status, number of sons, domestic housing structure, family environment, and family type. Husbands’ behavioral characteristics included personality type, playing cards/mahjong, gambling, smoking, and drinking. Smoking was defined as “continuous or cumulative smoking for six or more consecutive months” according to the WHO regulation, 1997. Drinking was defined as drinking once or twice a week or more. Consuming a small amount of alcohol during an occasional gathering was not defined as drinking. Playing cards/mahjong was defined as playing cards or mahjong one or more times in the past year. Age was categorized as 20–29, 30–39, 40–49, and ≥ 50 years. Education was categorized as primary school or less, junior high school, high school, junior college, and college or higher. Family types included a multigenerational family, a couple, a couple and a child, and a couple and children. The personalities of husbands was categorized as extrovert, middle type, and introvert, obtained by asking the women. If a husband was also interviewed, they judged their personality themselves. The occupations of the husbands were categorized as mental work, light manual labor, moderate manual labor, heavy manual labor, mental and physical labor, and unemployed. Monthly income was categorized as < 1000, 1000–1999, 2000–2999, and ≥ 3000 yuan. The variables of BMI, marital status, playing cards/mahjong, gambling, smoking, and drinking were dichotomized.

The Violence Against Women Instrument (VAWI), designed by the World Health Organization (WHO), was applied. This questionnaire was developed for use in different countries, including China, Bangladesh, Thailand, and other Asian countries, and was cross-culturally validated [20]. The scale comprises 20 items. Psychological violence was assessed with Items 1–5, physical violence was assessed with Items 6–11 and 15, and sexual violence was assessed with Items 12–14. If a respondent answered “yes” to any of the 15 items, the respondent was judged to have suffered DV by a current partner or any previous partner. Items 2–15 were used to assess whether the DV had occurred within 1 year or for a lifetime. The scale has been used in Shanghai and Fuzhou, China, for DV investigations [19, 21]. The VAWI has shown good internal reliability (α = 0.88) and construct validity (r = 0.89) [22].

### Sample size estimates

The incidence of DV in Wuhan was 28.32% [23]. The calculation formula for an epidemiological cross-sectional study sample size is n = tα^2^ × [P(1 − P)/d^2^], with α = 0.05, tα ≈ 2, and d = 0.1P, and the required sample size was 1013. Considering that there may be some invalid questionnaires, an additional 5% was added to the sample. A total of 1064 questionnaires were distributed in this survey, and 1015 were valid questionnaires, accounting for 95.4% of the questionnaires.

### Data analysis

Continuous variables were described as the mean ± standard deviation (SD) and tested with the rank-sum test; categorical variables were described as the proportion (%) and tested with the chi-square test or Fisher’s exact probability. Finally, multiple logistic regression was used to assess the association between lifetime DV and the characteristics of the husbands while adjusting for confounders. Separate models were run with each characteristic of the husbands as the dependent variable; lifetime DV was the primary independent variable, and age, marital status, playing cards/mahjong and gambling were confounding variables. The odds ratios (ORs) and 95% confidence intervals (CIs) were estimated from the multiple logistic regression models. All *P* values were two-tailed with a significance level of 0.05. Statistical analyses were carried out using SAS version 9.4 (SAS Inc., Cary, NC).

### Patient involvement

No patients were involved in developing the research question or the outcome measures, nor were they involved in planning the design, recruitment and conduct of the study. No patients were asked to advise on the interpretation or writing of the results. There are no plans to disseminate the results of the research to the study participants.

## Results

### The prevalence of DV

The prevalence of lifetime experience of violence was 29.36% (298/1015) and the prevalence of past-year violence was 12.20% (122/1015). Table 1 shows the prevalence of lifetime DV by the women’s and husbands’ characteristics. Women in the age group of 40–50 years (34.42%) and women whose husbands were in the age group of 40–50 years (34.00%) had a higher prevalence of lifetime DV than women in the other age groups. Lifetime DV was reported with a higher percentage of low literacy (52.38%) than with higher education (14.42%). Women with husbands who engaged in heavy physical labor (54.05%) were prone to have a higher prevalence of lifetime DV than women in the other groups. The prevalence of lifetime DV was greater among women with husbands who played cards (40.8%) and gambled (39.20%) than it was among nongamblers (27.75% and 27.98%). A higher proportion of smokers (33%) and drinkers (35.15%) reported lifetime DV compared to nonsmokers (23.89%) and nondrinkers (24.21%). Women whose husbands had short stature, were long-term smokers, and were long-term drinkers tended to have a higher prevalence of lifetime DV than those whose husbands had tall stature, were short-term smokers, and were short-term drinkers (*P* = 0.019, *P* < 0.001 and *P* < 0.001).

**Table 1:** The prevalence of lifetime DV by demographic characteristics

	No DV n (%)	DV n (%)	Chi-square (*Z*)	*P* value
Age, years			17.11	0.001
20–29	128 (83.66)	25 (16.34)		
30–39	213 (70.07)	91 (29.93)		
40–49	242 (65.58)	127 (34.42)		
≥ 50	134 (70.90)	55 (29.10)		
Spousal educational difference			0.03	0.984
Both equally educated	359 (70.39)	151 (29.61)		
Wife more educated	132 (70.97)	54 (29.03)		
Husband more educated	226 (70.85)	93 (29.15)		
Occupation			0.11	0.742
Employed	276 (70.05)	118 (29.95)		
Unemployed	441 (71.01)	180 (28.99)		
Marital status			7.71	0.006
First marriage	705 (71.28)	284 (28.72)		
Remarried/widowed/divorced	12 (46.15)	14 (53.85)		
The number of sons			15.29	0.001
0	300 (76.53)	92 (23.47)		
1	395 (67.99)	186 (32.01)		
≥ 2	22 (52.38)	20 (47.62)		
Family environment			10.04	0.018
Modern community	358 (74.74)	121 (25.26)		
Reconstruction of an urban village	39 (69.64)	17 (30.36)		
The old city	284 (65.59)	149 (34.41)		
Urban–rural fringe	36 (76.60)	11 (23.40)		
Family type			12.42	0.015
Multigenerational family	194 (73.76)	69 (26.24)		
A couple	60 (67.42)	29 (32.58)		
A couple and a child	384 (73.14)	141 (26.86)		
A couple and more than one child	79 (62.20)	48 (37.80)		
Domestic housing structure			13.14	0.004
Self-built	22 (53.66)	19 (46.34)		
One room and one hall	50 (60.24)	33 (39.76)		
Two rooms and one hall	409 (70.88)	168 (29.12)		
Three rooms and one hall	236 (75.16)	78 (24.84)		
Age of the husband, years			14.57	0.002
20–29	93 (84.55)	17 (15.45)		
30–39	193 (71.75)	76 (28.25)		
40–49	264 (66.00)	136 (34.00)		
≥ 50	167 (70.76)	69 (29.24)		
Education of the husband			23.95	< 0.001
≤ Primary school	10 (47.62)	11 (52.38)		
Junior high school	204 (64.76)	111 (35.24)		
High school	275 (70.33)	116 (29.67)		
Junior college	139 (75.54)	45 (24.46)		
≥ College	89 (85.58)	15 (14.42)		
Occupation of the husband			25.19	0.001
Mental work	157 (79.70)	40 (20.30)		
Light manual labor	72 (72.73)	27 (27.27)		
Moderate manual labor	255 (65.89)	132 (34.11)		
Heavy manual labor	17 (45.95)	20 (54.05)		
Mental and manual labor	191 (74.32)	66 (25.68)		
Unemployed	25 (65.79)	13 (34.21)		
Income/m of the husband, yuan			7.09	0.312
< 1000	23 (63.89)	13 (36.11)		
1000–1999	46 (67.65)	22 (32.35)		
2000–2999	183 (69.85)	79 (30.15)		
3000–3999	210 (71.67)	83 (28.33)		
4000–4999	93 (68.89)	42 (31.11)		
≥ 5000	135 (76.70)	41 (23.30)		
Do not know	27 (60.00)	18 (40.00)		
BMI of the husband			3.89	0.049
Normal or underweight	532 (72.38)	203 (27.62)		
Overweight or obese	185 (66.07)	95 (33.93)		
Personality of the husband			2.34	0.310
Extrovert	7 (58.33)	5 (41.67)		
Middle type	641 (71.38)	257 (28.62)		
Introvert	69 (65.71)	36 (34.29)		
Playing cards/mahjong			9.00	0.003
No	643 (72.25)	247 (27.75)		
Yes	74 (59.20)	51 (40.80)		
Gambling			6.66	0.010
No	641 (72.02)	249 (27.98)		
Yes	76 (60.80)	49 (39.20)		
Smoking			9.76	0.002
No	309 (76.11)	97 (23.89)		
Yes	408 (67.00)	201 (33.00)		
Drinking			14.59	< 0.001
No	407 (75.79)	130 (24.21)		
Yes	310 (64.85)	168 (35.15)		
Height (cm)	172.30 ± 5.17	171.60 ± 5.10	− 2.34	0.019
Weight (kg)	69.41 ± 9.24	70.47 ± 10.37	0.95	0.341
Smoking, years	8.99 ± 10.13	12.49 ± 11.28	4.63	< 0.001
Drinking, years	6.83 ± 9.42	9.97 ± 10.68	4.71	< 0.001
Total	717 (70.64)	298 (29.36)		

### The prevalence of all kinds of lifetime DV

All kinds of lifetime DV are shown in Table 2. The prevalence of psychological violence (28.28%) was the highest among all kinds of lifetime DV. The forms included neglect, insults, threats, and so on. The prevalence of physical violence was the second highest; the prevalence was 6.6%. It included slapping, pulling hair, punching, etc. The prevalence of sexual violence (3.55%) was the lowest and included compulsory actions.

**Table 2:** The prevalence of all kinds of lifetime DV

DV category	*n*	%
Physical violence	67	6.60
Slapping/throwing things	52	5.12
Pushing/pulling hair	46	4.53
Punching	35	3.45
Kicking/dragging/beating	33	3.25
Choking/burning	2	0.20
Threatened to use a knife	2	0.20
Psychological violence	287	28.28
Preventing seeing friends	12	1.18
Limiting contact with family	9	0.89
Always wanting to know where his wife is	60	5.91
Neglect/indifference	175	17.24
Being angry with his wife if she talks with other men	32	3.15
Doubting fidelity	8	0.79
Needs husband permission to go to the hospital	21	2.07
Insults	125	12.32
Putting wife in front of others	28	2.76
Intimidation by some behavior	119	11.72
Threatening to harm someone his wife cares about	16	1.58
Sexual violence	36	3.55
Compulsory actions	32	3.16
Does not want to have sex since she is afraid of her husband	9	0.89
Allows wife to feel shame about sex	9	0.89

### Association between lifetime DV and husbands’ characteristics

The associations between lifetime DV and the characteristics of the husbands are shown in Table 3. After adjusting for possible confounding variables, lifetime DV was more likely to be reported for hard physical laborers (OR 3.54, 95% CI 1.63–7.77) and drinkers (OR 1.60, 95% CI 1.19–2.14). Long-term smoking was associated with increased odds of lifetime DV (OR 1.03, 95% CI 1.01–1.04) and being overweight or obese was associated with increased odds of lifetime DV (OR 1.36, 95% CI 1.01–1.88). A high education level was associated with decreased odds of lifetime DV (OR 0.80, 95% CI 0.66–0.96).

**Table 3:** Multiple logistic regression models for the relationship between lifetime DV and the characteristics of husbands

Factor	Estimate of parameter	Standardization of parameter estimates	Chi-square	*P* value	A*OR* (95% CI)
Husband’s education	− 0.225	− 0.096	5.490	0.019	0.80 (0.66–0.96)
Husband’s occupation					
Heavy physical labor: Mental work	1.247	0.127	9.998	0.001	3.54 (1.63–7.77)
Husband’s years spent drinking	0.465	0.129	9.597	0.002	1.60 (1.19–2.14)
Husband’s BMI					
Overweight or obese: Normal or underweight	0.356	0.162	4.828	0.028	1.36 (1.01–1.88)
Husband’s years spent smoking	0.033	0.135	10.062	0.002	1.03 (1.01–1.04)

Adjusting for the women's ages, spousal educational difference, women's occupations, marital status, number of sons, family environment, family type, and domestic housing structure

## Discussion

The study aimed to understand the prevalence of DV in Wuhan, China, and to explore the correlation between lifetime DV and husbands’ characteristics.

Women whose husbands had lower education, performed heavy manual labor, were long-term alcohol drinkers, had overweight or obesity, and were long-term smokers were prone to lifetime DV. Women with more educated husbands had a lower risk of suffering from lifetime DV, a finding that was consistent with previous studies [15, 18, 24, 25]. The lower the education levels of the husbands, the more likely they were to use violence to solve problems. The low degree of culture was usually on behalf of a husband’s low social and economic status. These factors all easily led to family violence.

This study indicates that women whose husbands perform heavy physical labor were prone to lifetime DV, which is consistent with previous studies [26, 27]. Compared with those who perform mental labor, men who perform heavy manual labor prefer to use a simple and arbitrary manner to solve family conflicts, and negative emotions caused by job burnout may be brought to the home, including failing to care about their wives and irritability, resulting in the occurrence of DV.

Our results are also in accordance with previous research that has suggested that lifetime DV is more likely to be reported for husbands who binge drink [28, 29]. As a mood enhancer, alcohol can enhance a person's sense of anger and frustration. Binge drinkers may be dependent on alcohol, lack communication with their family, and receive less family support. Therefore, these factors may easily lead to the occurrence of family conflicts. Tedor et al. [30] reported that alcohol dependence has an obvious correlation with violent attacks. This also suggests that the control of alcohol consumption and the frequency of alcohol intoxication are very important for the prevention and control of DV.

After searching the PubMed, China Academic Journals (CNKI), and Wanfang databases, it was determined that the correlation between body mass index and DV is rare in DV-related factor research at present. This research showed that the physical build of husbands and the close degree of obesity were associated with lifetime DV, but the reason remains to be further researched. This result was supported by previous research that suggested that women who experienced higher levels of violence preferred the faces of men with low BMI [31].

Smoking by husbands is a risk factor for lifetime DV. Smoking is considered to be a way to relieve stress. However, heavy smokers rely on smoking to resolve anguish and form a kind of depressive personality. Once this personality is developed, it can lead to DV. Previous studies have reported similar associations [32, 33].

In summary, the prevalence of lifetime DV in women from Wuhan, China, was common. We might decrease the occurrence of the possible risk factors for lifetime DV to control it. There was a strong correlation between lifetime DV and a husband's characteristics. Lifetime DV was related to a husband's cultural level, professional career, alcohol and tobacco habits, and body mass index. Women whose husbands had lower education, performed heavy physical labor, were long-term drinkers, had overweight or obesity, and were long-term smokers were vulnerable to lifetime DV. Family violence prevention is needed to improve husbands’ cultural quality and poor living habits. In future work, we need to explore the risk or protective factors for past year violence.

The limitations of this study include the following aspects. First, as a result of the limitation of conditions, the questionnaire survey was only carried out in a community health service center. This limits the extrapolation of the conclusions. Second, this study was a cross-sectional survey, and the occurrence of lifetime DV and the husbands’ characteristics was obtained at the same time, so it is impossible to judge the causal relationship between them. Finally, some of the items in the scale were sensitive, some respondents had difficulties when answering the questions, and the truth of the answer was not guaranteed.

## Data Availability

The datasets generated and/or analyzed during the current study are not publicly available due to institutional policy but are available from the corresponding author on reasonable request.
